# Excessive generalization of pain-related avoidance behavior: mechanisms, targets for intervention, and future directions

**DOI:** 10.1097/j.pain.0000000000002990

**Published:** 2023-07-27

**Authors:** Kristof Vandael, Bram Vervliet, Madelon Peters, Ann Meulders

**Affiliations:** a Experimental Health Psychology, Maastricht University, Maastricht, the Netherlands; b Laboratory of Biological Psychology, KU Leuven, Leuven, Belgium; c Research Group Health Psychology, KU Leuven, Leuven, Belgium

## 1. Introduction

Avoidance of pain-associated activities is adaptive in acute pain because it prevents harm; for example, when shooting pain is experienced while bending, not repeating this movement may prevent exacerbating an injury. However, when tissues have healed, avoidance prevents learning that these activities are actually safe. Furthermore, avoidance can spread toward activities similar to those previously associated with pain, even if these were never paired with pain (avoidance generalization^21^). This again is adaptive because a learned protective behavior can be applied to similar instances, without needing to learn about each separately. However, when applied to safe activities, it bears the risk of increased withdrawal from harmless daily activities. According to contemporary fear-avoidance models, such excessive generalization (or overgeneralization) may instigate a self-sustaining cycle of activity disengagement, resulting in chronic pain disability.^8,35,41,82^

Because of the central role of avoidance in chronic pain disability, gaining insight into factors that tackle its excessive generalization can help to develop and optimize interventions reducing pain-related suffering. According to fear-avoidance models, pain-related fear can initiate avoidance behavior intended to avert bodily threat. Therefore, we first review experimental studies on (over)generalization of pain-related fear before moving to avoidance. Next, targets for intervention are discussed considering empirical evidence from both the field of chronic pain and anxiety disorders. Note that the current review focuses on behavioral interventions and excludes, for example, neural stimulation methods.^6^

## 2. Generalization of pain-related fear

Pavlovian conditioning plays a key role in learning to predict potential threats such as bodily harm.^41^ Pain is a biologically salient signal that indicates harm, making it an important motivator for learning. Experimental studies show that an initially neutral movement paired with pain comes to elicit pain-related fear.^30,42,48^ Fear can then spread towards movements resembling the pain-associated movement, although they were never paired with pain (stimulus generalization^10,37,47,49^). Movements that are less similar to the pain-associated movement typically elicit less fear. In addition, pain-related fear can spread towards perceptually similar contexts and along a dimension of conceptual relatedness, ie, pain-related fear can spread towards movements that have the same function or belong to the same category as the pain-associated movement (eg, gardening^20,43,46^).

From an evolutionary perspective, generalization is adaptive because we do not need to learn everything anew: extrapolating threat value to new stimuli based on (perceptual) similarity means that we do not have to learn about each from experience but can generate defensive responses based on previous learning. However, this may become maladaptive when stimuli bearing only minimal similarity to the original threat-associated stimulus elicit fear, leading to false positives (ie, signaling threat when there is none). Such overgeneralization is considered a transdiagnostic pathogenic marker in anxiety disorders,^9,38,39^ and recently, the same argument has been made for chronic pain disorders.^41,42,82^ For example, when someone is afraid of slightly bending the back, this is maladaptive if they originally experienced pain during a very different movement—such as bending over a 90-degree angle while lifting a heavy object. Experimental studies indeed showed overgeneralization of pain-related fear in various chronic pain conditions compared with healthy, pain-free controls, corroborating the role of overgeneralization in chronic pain.^24,44,45^

## 3. Generalization of pain-related avoidance

Next to pain-related fear, contemporary fear-avoidance models emphasize the role of avoidance in the development and maintenance of chronic pain disability.^82^ Avoidance can be particularly disruptive in daily life and is self-sustaining—it prevents learning that activities are safe. Avoidance can be acquired through operant conditioning: people learn that certain behaviors or movements lead to the nonoccurrence of a feared event such as pain.^19,78^ This learning strengthens avoidance behaviors, making them more likely to occur. Besides the nonoccurrence of the feared event, other factors have been argued to reinforce avoidance, such as fear reduction and relief—the positive feeling in reaction to the absence of an anticipated aversive event.^34,54,80^ Importantly, avoidance of pain-associated movements can generalize toward perceptually similar movements.^21^ This is again adaptive from an evolutionary perspective because it reduces the chance of encountering threats such as bodily harm. However, it becomes maladaptive when applied excessively, as this can interfere with daily functioning and valued activities (eg, playing with children) and reduce physical activity. The tipping point where adaptive generalization becomes maladaptive is often elusive, and behaviors that may have been adaptive in the past—eg, with severe tissue damage—may become maladaptive when they are not updated based on sensory information or environmental circumstances.^73^

Currently, experimental studies on overgeneralization of avoidance in chronic pain conditions are lacking and scarce in clinical anxiety.^64^ Contemporary emotion theories consider avoidance as a component of fear because emotions are viewed as a compound of cognitions, action tendencies, physiological responses, motor actions, and subjective feelings.^34,50^ This means that overgeneralized fear is likely to be accompanied by overgeneralized avoidance, but there is no one-to-one relationship between them, emphasizing the need for research on overgeneralization of avoidance specifically.^18,65^ For example, although avoidance behaviors are strengthened when a feared outcome does not occur, they can simultaneously be weakened by costs—such as not being able to participate in valued activities. This can lead to fear and avoidance dissociating because activities are performed despite fear.^7,74^

**Figure 1.**
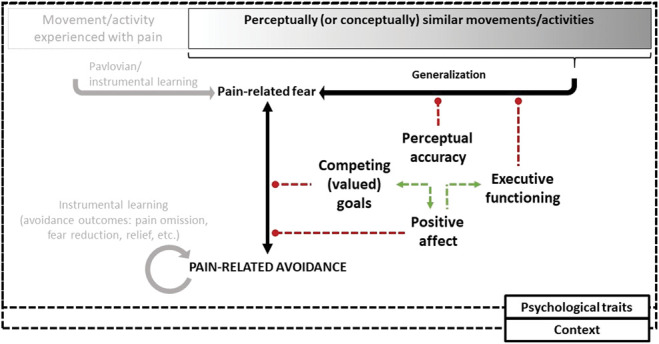
Theoretical framework: Dotted red lines show hypothesized attenuating effects on fear generalization and/or the fear-avoidance relation. Dotted green lines show hypothesized enhancing effects between intervention targets. Context and psychological traits are considered to modulate fear generalization and the fear-avoidance relation (eg, more fear generalization in threatening contexts/highly anxious individuals), as well as the effect of interventions (eg, stronger attenuating effects in safe contexts). Context and traits are also considered to have interacting effects (eg, stronger association between trait anxiety and fear generalization in threatening contexts). Note that the acquisition of pain-related fear and the reinforcement of avoidance behavior through associative learning are not the focus of intervention in the current review and are therefore displayed in gray.

Both from a theoretical and clinical perspective, it is important to gain insight into ways to reduce overgeneralization of fear and associated avoidance. We identified several potential intervention targets to reduce avoidance overgeneralization: goals competing with avoidance, perceptual accuracy, and positive affect. For each factor, we review empirical evidence supporting that intervention affects fear generalization and/or the fear-avoidance relation in humans (Fig. 1). We also discuss other potential factors to intervene on, based on observational studies indicating their implication in fear generalization and/or the fear-avoidance relation.

## 4. Intervention targets to attenuate avoidance generalization

### 4.1. Competing goals

As avoidance occurs in a dynamic environment of concurrent, potentially competing, goals, the motivational context should be taken into account.^74^ Some individuals prioritize controlling pain at the cost of competing valued goals, instigating the vicious cycle of disability described by fear-avoidance models.^82^ Goals are the focus in certain psychological interventions for chronic pain.^55^ For example, acceptance and commitment therapy encourages participants to identify and pursue valued goals and can reduce the extent to which pain interferes with daily functioning.^83^ Experimental studies in both the field of anxiety and pain indeed show that the presence of competing goals (eg, obtaining monetary reward) attenuates avoidance.^7,61,62^ Such goals do not necessarily reduce fear directly but rather disrupt the fear-avoidance relation.^61^

Based on these findings, an operant-based approach in which behaviors competing with avoidance are reinforced could be applied to weaken the relation between overgeneralized fear and avoidance. This idea is supported by experimental work from the anxiety field; reinforcing responses that are incompatible with avoidance leads to reduced generalization of conditioned avoidance toward conceptually related stimuli.^1^ Whether reinforcement of alternative behaviors and activities attenuates generalization of pain-related avoidance in people who prioritize pain control remains to be investigated; reinforcements may not be experienced as rewarding or motivating to the same extent for everyone,^60,63^ and altered reward responsivity has been observed in people with chronic pain.^67^

### 4.2. Perceptual accuracy

Perceptual inaccuracy may lead to overgeneralization of protective responses because generalization negatively relates to the degree to which one stimulus can be differentiated from another.^15,52,69,86^ For example, when movements that were never paired with pain are not accurately perceived, they may be more likely to elicit pain-related fear and avoidance. This implies that perceptual discrimination training reduces generalization. Experimental studies in the anxiety field show that training healthy participants to differentiate between visual stimuli (eg, shapes) indeed leads to less generalization of conditioned fear.^17,26^ Moreover, such visual discrimination training has also been shown to attenuate generalization of avoidance, both in healthy participants^40^ and participants with subclinical anxiety.^16^

In the context of pain, somatosensory and proprioceptive information is as important as visual information for fear and avoidance learning and subsequent generalization.^77^ Studies indeed show that tactile acuity—the accuracy of sense of touch—affects pain-related fear learning.^23^ Moreover, improving tactile acuity reduces pain intensity in chronic pain conditions.^53^ When learning about movements specifically, proprioceptive information plays a key role, ie, the perception of motion and position of the body (segments) in space.^66^ A wide range of pain conditions present with impaired proprioceptive accuracy,^29,32,68,72^ and evidence suggests that targeting this specific impairment may improve pain outcomes.^28^ Moreover, an experimental study showed an association between poor proprioceptive accuracy and excessive avoidance of pain-associated movements in pain-free participants, suggesting that proprioceptive training is indeed a pathway to counter overgeneralization of pain-related avoidance.^79^

### 4.3. Positive affect

Fear-avoidance models stress the importance of vulnerability factors such as negative affect in chronic pain disability. In addition, evidence for the role of resilience factors such as positive affect has accumulated.^12,22,57,70^ Studies in people with chronic pain show that positive affect may be depleted during pain, and that positive affect inversely predicts pain reports.^87,88^ Positive psychology interventions have been shown to successfully promote positive affect, wellbeing, and functioning and reduce pain severity and depression in individuals with chronic pain (see Ong et al.^56^ and Braunwalder et al.^5^ for systematic reviews).

Evidence suggests that positive affect facilitates learning that certain stimuli are safe and thus inhibits fear from spreading to novel safe stimuli.^89^ Geschwind et al.^14^ showed that experimentally induced positive affect was indeed associated with less generalization of pain-related fear toward movements similar to a safe movement, whereas generalization toward movements similar to a pain-associated movement was preserved. This makes sense because it is adaptive to reduce the chance of encountering threat by generalizing fear toward stimuli resembling a threat-associated stimulus, whereas it is maladaptive to increase false positives by generalizing fear toward stimuli resembling a safety-associated stimulus. Furthermore, positive affect may increase willingness to approach fear-evoking stimuli (ie, to not avoid them), thus potentially affecting the fear-avoidance relation as well.^89^

### 4.4. Further potential intervention targets

Executive functions such as working memory and attentional control are also impaired in chronic pain conditions, indicating potential for intervention.^2,71^ Evidence confirms that working memory plays a role in generalization, although research attempting to experimentally improve working memory to attenuate generalization is lacking.^36,84^ Interventions targeting executive functioning have already shown promise in the field of depression^33^ and may be translated for use in the pain field. Intriguingly, inducing positive affect improves executive functions, including working memory, indicating that cognitive processes may mediate the effect of positive affect induction on generalization.^3,4,85^

Anxious traits such as anxiety, sensitivity, and intolerance of uncertainty are associated with more fear generalization and with a stronger fear-avoidance relation, indicating another avenue for intervention.^27,51^ Treatments targeting such traits have been developed in the anxiety field,^76^ and translation may prove fruitful in the pain field. Furthermore, traits may help identify at-risk individuals, who potentially benefit most from interventions. For example, individuals with high intolerance of uncertainty may benefit from proprioceptive accuracy training to reduce uncertainty about movements.

## 5. Future directions

Although paradigms have been developed to study generalization of avoidance behavior in pain research,^21^ diagnostic and predictive validity still needs to be established.^81^ In other words, we need evidence that patients with chronic pain show overgeneralization of avoidance in these paradigms, and that such overgeneralization is linked to reduced functioning in daily life (Table 1). Next, validated paradigms can be used to test experimental interventions, both in healthy subclinical and clinical samples. All discussed intervention targets need investigation in the context of pain; experimental research showing that these causally affect generalization of pain-related avoidance is needed. The listed interventions theoretically could be applied to counter overgeneralization along a perceptual dimension (ie, perceptually similar stimuli/responses/contexts) and a conceptual dimension. It is counterintuitive to train perceptual accuracy to reduce generalization along a conceptual dimension. However, from a predictive processing perspective, fear overgeneralization results from giving more weight to the affective-motivational aspects of input at the expense of detailed sensory-perceptual input.^75^ Training proprioceptive accuracy may lead to increased weighing of sensory-perceptual input and decreased weighing of affective-motivational aspects, meaning less emphasis on inferences based on conceptual relationships, thus attenuating overgeneralization.

**Table 1:** Future directions.

Methodology	Research question	Population	Relevance
Experimental paradigms	Do patients generalize their pain-related avoidance more compared with pain-free controls? • Along perceptual and conceptual dimensions? • Along contexts (ie, reduced context modulation)?	Patients with chronic pain vs. pain free controls	Diagnostic validity paradigm
	Is more generalization of pain-related avoidance in experimental paradigms associated with reduced functioning in daily life? • At same time point (avoidance generalization as a maintaining factor)? • In future (avoidance generalization as an instigating factor)?	Patients with chronic pain	Predictive validity paradigm
	Does manipulation of hypothesized intervention targets lead to less generalization of pain-related avoidance? • Before avoidance conditioning (modelling prevention)? • Before/between generalization test(s) (modelling intervention)?	Pain free/subclinical* & patients with chronic pain	Proof-of-concept/predictive validity paradigm Proof-of-concept/predictive validity paradigm
Single-case experiments/randomized controlled trials	Improved functioning in daily life after interventions directed at the hypothesized intervention target? • Using prevention or intervention strategies informed by/translated from experimental manipulations (potentially as add-ons to treatments); eg, proprioceptive accuracy training could be implemented in a virtual reality task performed in the home environment; • When matching interventions to specific risk factors (“what works for whom”)? eg, low levels of positive affect may be an indication for positive psychology interventions.	Patients with chronic pain	Proof of concept

* For example, selecting participants (without chronic pain) on specific traits, such as (high) pain-related fear and (low) positive affect. Diagnostic validity refers to the extent that behavior in the paradigm differs between patients and pain-free controls; predictive validity refers to the extent that behavior in the paradigm predicts behavior in daily life—eg, if a manipulation models a therapeutic intervention known to affect behavior in daily life, evidence for a significant effect on behavior in the experimental paradigm contributes to the predictive validity of the paradigm.

As contemporary models of chronic pain recognize the importance of social factors next to biological and psychological ones,^13^ the direct social context and the wider sociocultural context in which generalization occurs—and/or developed—deserve scrutiny. Experimental research showed that a threatening social context facilitates the acquisition of pain-related fear,^31^ but the role in generalization remains underinvestigated. Furthermore, our review focused on experiential learning, whereas pain-related fear and avoidance can also be acquired and generalized based on observational or instructed learning^11,25^; these mechanisms deserve attention as well.

Investigation of experimental interventions to counter overgeneralization is crucial to inform and strengthen evidence-based treatment. Goal-directed interventions,^83^ proprioceptive accuracy training,^28^ and positive psychology interventions^59^ have already been implemented—to varying extents—as clinical treatments in chronic pain, and other existing interventions may unintendedly use the discussed mechanisms (eg, overcoming fear during exposure therapy may lead to positive affect). Experimental studies can provide insights into underlying mechanisms of such interventions and ways to optimize treatments or identify novel targets for treatment. Furthermore, the preventive potential of interventions in the acute pain stage remains underinvestigated. The experimental model should reflect whether the interest is in prevention before or during the acute stage (eg, before/after surgery) or in treatment during the chronic stage. For example, interventions can be inserted before conditioning to model the former^40^ or in between generalization tests to model the latter.^26^

Once experimental research provided insight into the relevant mechanisms, experimental interventions can be translated into practice to test for clinically relevant improvements. Translation can occur in a variety of ways; proprioceptive accuracy training could, for example, be implemented in a virtual reality task performed in the home environment. Such tasks could then be evaluated (as add-ons to existing treatments) using single-case experimental designs.^58^ Figuring out “what works for whom” may be crucial because patients present with specific problems (and idiosyncratic learning histories): eg, poor proprioceptive accuracy may be an indication for proprioceptive training, whereas reduced reward responsivity may be a counter indication for goal-directed approaches. Indeed, people with chronic pain show altered reward responsivity, resulting in limited reward learning.^67^ Furthermore, intervention aspects may be combined to improve outcomes: eg, positive psychology interventions may be used to motivate physical exercise to increase perceptual accuracy or the performance of valued activities in goal-directed approaches.

In summary, experimental paradigms to study pain-related avoidance generalization have already been developed and now need to be applied to chronic pain samples to demonstrate excessive avoidance generalization. Next, these paradigms can be used to test potential intervention targets to reduce avoidance generalization: first in pain-free samples as a proof of concept and then in chronic pain samples. We argued that goals competing with avoidance, perceptual accuracy, and positive affect are promising targets. Ultimately, such research can form the basis to develop and improve clinical treatments.

## Conflict of interest statement

The authors have no conflicts of interest to declare.
